# Assessing the Attitude of Tampa Bay Youth toward HIV Self-Testing Kits

**DOI:** 10.3390/tropicalmed6030111

**Published:** 2021-06-25

**Authors:** Sherry Zhang, Isabella Lopez, Bernard Washington, Brittney Gaudet, Carina A. Rodriguez, Lisa J. Sanders

**Affiliations:** 1 Morsani College of Medicine, University of South Florida, Tampa, FL 33612, USA; sherryz@usf.edu (S.Z.); lopezi@usf.edu (I.L.); bgaudet@usf.edu (B.G.); 2 Division of Pediatric Infectious Diseases, Morsani College of Medicine, University of South Florida, Tampa, FL 33606, USA; bwashington@usf.edu (B.W.); crodrig1@usf.edu (C.A.R.)

**Keywords:** youth, adolescents, HIV testing, attitude, self-testing

## Abstract

In adults, data support the utility and acceptance of home HIV testing; however, in youth, particularly in the US, this has not been well studied. In this exploratory study, we surveyed Tampa Bay youth aged 16−27 and attending sexual health clinics between 1 June and 31 June 2018 (*n* = 133) regarding attitudes and perceptions towards HIV self-testing. While most indicated the clinic over home when asked for preferred testing location, study population and subgroup analysis demonstrated a positive response (agree) to Likert-scale questions regarding the use of home HIV self-testing kits and negative responses (strongly disagree) to “would not use self-testing kit”. There was a significant difference between genders in testing location preference (*p* = 0.031) for those respondents that specified gender (*n* = 123), with males more likely to prefer home testing than females. This study suggests an openness of youth towards HIV home testing that could help to expand the number of youth aware of their HIV status.

## 1. Introduction

Youth and young adults are a priority target population in terms of HIV diagnosis and prevention, as they represent 21% of all newly diagnosed HIV cases in the United States (US) [1,2]. The Centers for Disease Control’s HIV Surveillance Report from 2020 demonstrates that while the overall rates of HIV diagnosis for persons aged 13−24 decreased from 2014 to 2018, the rate for ages 20−24 remained the second-highest rate of all age brackets at 27.9 people per 100,000 [1]. High infection rates and a lack of knowledge of infection status are important issues to address when working to prevent the continued spread of HIV in young people. In 2018, of the estimated 47,800 youth living with HIV in the United States (US), only about half were aware of their infection status [3]. For male adolescents that have sex with other males, only 11.5% have been tested for HIV [4]. In addition, youth with HIV are the least likely of any age group to be retained in care or to have a suppressed viral load, thereby increasing the risk of transmission to their sexual partners.

Compounding a lack of knowledge of HIV status, studies have documented numerous other health-related prevention challenges in youth. Nationwide, surveys of sexually active high school students show that 46.2% say they did not use condoms during their last sexual intercourse [4]. Of male students who have sex with men, 13.5% confirmed having had four or more sexual contacts in their lifetime, increasing the likelihood of HIV transmission [4]. About 20% of sexually active students drank alcohol or reported other substance use prior to their most recent sexual intercourse, leading to a greater likelihood of having sex without protection [4]. Youth are also less likely to use pre-exposure prophylaxis as a prevention strategy for HIV due to barriers including cost, access, and stigma [1]. Finally, various socioeconomic challenges for youth such as low-income households, homelessness, incarceration, and being uninsured contribute to lack of access to HIV testing and care and make viral suppression difficult [1].

Routine HIV testing is important in maintaining the sexual, reproductive, and general health of patients. It is well recognized that HIV testing is foundational to HIV prevention strategies, yet barriers to the widespread testing of youth remain. Estimates suggest that only 22% of high-school-aged youth have been tested, a number that remained virtually unchanged from 2005 to 2013 despite recommendations for routine screening [5]. A recent study of youth showed that only about 60% of youth having sexual activity with a positive partner had been tested for HIV or knew their status, while only 40% of youth knew the HIV status of their sexual partner [6]. Common reasons youth cite for not being tested include never having been offered testing, not thinking they could be HIV positive, being upset at the idea of testing positive to find out their status, not wanting anyone to find out their test results, and difficulties with the testing process [7]. In-home HIV self-testing for youth and/or their partners is one possible way to overcome some of these barriers and increase youth testing rates. 

In adult populations, the literature supports the idea that in-home HIV testing increases testing rates, leads patients to seek appropriate care, and bolsters prevention efforts [8,9]. However, the acceptability and outcomes of HIV self-testing interventions in US youth have not been well studied. Specifically, it is not known if the benefits observed in adults regarding prevention and linkage to care can be replicated in US youth. The recent COVID-19 pandemic and subsequent worldwide lockdowns led to increased concerns about the availability of HIV testing and HIV/AIDS treatment. In response, the World Health Organization recommended HIV self-testing as an additional method to increase HIV testing coverage and frequency [10]. Studies in Sub-Saharan Africa during these COVID-19 lockdowns found a high acceptance rate of HIV self-testing across various demographics; however, these studies did not include anyone younger than 18 [10]. The purpose of this study was to evaluate the efficacy and feasibility of implementing in-home HIV testing for US youth by surveying youth attitudes regarding this type of testing, with the intent to provide a foundation for future inquiries into the role of HIV self-testing among youth populations.

## 2. Materials and Methods

### 2.1. Study Population, Variables, and Design

Our observational cross-sectional survey study was conducted on youth between the ages of 16 to 27 years old presenting to four clinics associated with the University of South Florida (USF) Department of Pediatrics. Clinics included the Ybor Youth Clinic, a storefront clinic providing youth sexual health services to under-resourced youth located in a high HIV-incidence zip code; two pediatric HIV Clinics, one at St. Petersburg’s All Children’s Hospital and the other at the Children’s Medical Service Lakeland Clinic; and USF Student Health Services, which provides on-campus health service to university students. Youth who presented to any of these clinics for HIV or STI testing or who were known to be HIV positive were given the opportunity to participate in the study. Survey participation was voluntary and verbal consent to participate was obtained by study personnel. Patients were informed that their answers were confidential, anonymous, and would not influence their course of care. The paper-based surveys exploring attitudes and perceptions about HIV self-testing were distributed during the clinic check-in process or during the risk-reduction portion of rapid HIV-testing visits. Data were collected from 1 June to 31 July 2018 and transcribed from the paper survey to an electronic file by study personnel. No identifiable information was collected or stored. 

The survey consisted of 25 questions regarding patient demographics, sexual history, opinions on HIV self-testing, and perceived limitations to self-testing. Demographic questions included age, gender, sexual history, and STI/HIV history. One question specifically asked where they would prefer to have HIV testing done, in a clinic or at home. Other attitudes and preferences towards HIV self-testing (e.g., “I would use an in-home, HIV self-testing kit if provided and explained to me by my doctor”, “I would encourage my partner or partners to contact or see a doctor if their result of an in-home test was positive for HIV”) were rated on a 5-point Likert scale from 1 (strongly disagree) to 5 (strongly agree). 

### 2.2. Statistical Analysis

Patients with unknown or undetermined data for certain categories and responses were excluded from univariate analyses for those respective variables. The dataset was subdivided into subsets based on gender, previous HIV testing, age, previous STI testing, previous positive STI history, and previous experience performing a self-test. Differences in continuous variables were assessed using Student’s t test with Cohen’s d as the calculation for the effect size [11]. Analysis of differences between demographic groups regarding in-home versus clinic testing was performed with chi-square tests of independence. A Fisher’s exact test was utilized in cases where *n* < 5 and effect size was determined using Phi (φ), given that the analysis utilized 2 × 2 contingency tables [12]. Likert scale results were compared against demographic groups and analyzed using an independent-samples t test. Statistical significance was defined as *p* ≤ 0.05 for each statistical test, without adjustment for multiple testing. Data were analyzed using SPSS Version 24 (IBM Corporation, Armonk, NY, USA).

## 3. Results

Of 143 surveys that were conducted, 10 without Likert rankings about attitudes and preferences towards HIV self-testing were excluded (5 females, 5 males, average age 21.9). Of the remaining 133 surveys, participants were not required to respond to all of the other questions for their results to be analyzed. Regarding the number of participants from each clinic site, 121 completed surveys at the Ybor Youth Clinic, 2 surveys were completed at each of the HIV clinics, and 8 surveys were completed at Student Health Services. Participants were 21.2 years old on average, with a standard deviation of 2.3 years. Ages ranged from 16 to 27 years old. Seven participants were under the age of 18. A total of 63 (51.2%) of participants who specified a gender identified as female. Eight respondents left gender blank and two misinterpreted the question and responded with their sexual orientation rather than gender. Of the 133 youth surveyed, 97 (72.9%) reported ever having been tested for HIV and, of the 97 patients whose HIV status was known, 11 reported being HIV positive. A total of 70.2% reported having undergone STI testing. Of those that had received STI testing previously, 52.9% had tested positive for either gonorrhea or chlamydia. Characteristics of the 133 patients whose surveys were analyzed are summarized in Table 1. 

### 3.1. Preferred Location and Method

**Figure 1:**
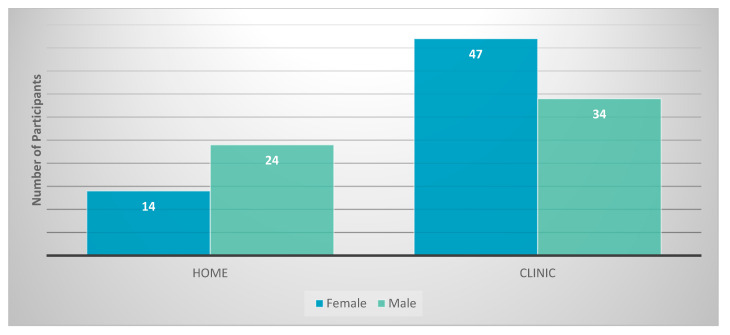
Testing location preference by gender.

Regarding preferred testing location (home versus clinic) 38 participants preferred testing at home versus 81 who preferred testing in a clinic (Figure 1). Four respondents did not have a preference. 

Of the youth who specified a gender (*n* = 123), there was a significant difference between genders in preference for testing location. Males were more likely than females to prefer testing at home, but both males and females expressed an overall preference for clinic testing. There was no statistical difference associated with previous HIV testing, previous STI testing, history of a previous STI, and previous use of an HIV self-testing kit and preference for testing location. Age was not statistically associated with a preference for testing location (Table 2). Of the 11 youth surveyed who were known to be HIV positive, none had ever used a self-test test kit. Four preferred at-home testing and seven preferred testing in a clinic setting, reflecting attitudes similar to those of the other respondents.

### 3.2. Attitudes Towards Testing Use

**Figure 2:**
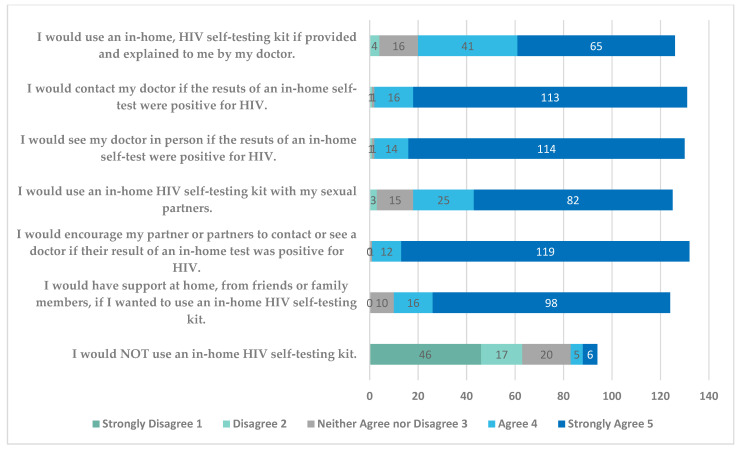
Likert-Scale Responses for HIV Self-Testing at Home Questions.

Figure 2 shows the statements that youth responded to and the Likert-scale responses to the home-testing questions included in the survey. The results show that a large majority of participants were open to the idea of in-home testing, with average responses in the 4–5 range (“agree” to “strongly agree”) for the statements relating to using an in-home HIV self-testing kit, seeing a doctor regarding results, encouraging use of kits with partners, and being supported in using an in-home test. Ten participants did not respond to the last question on the survey regarding not using an in-home test and 29 were not sure of their response. However, of those that responded, 63/94 (67%) disagreed or strongly disagreed with the statement that they would not use an in-home HIV test. 

There were no statistically significant differences (*p* > 0.05) between the various demographic groups (gender, previous HIV testing, previous STI testing, and ever STI positive) and responses to the Likert-scale questions regarding attitudes about HIV self-testing at home. HIV-positive patients were analyzed separately due to their positive status and possible differences in interpretation of the attitude questions, leading to measurement invariance. When doing subgroup analysis of the HIV-positive group, similar results were found regarding the Likert-scale questions. They averaged in the 4s in regard to openness to home testing, and in the 1s in response to not using an in-home HIV self-testing kit. 

## 4. Discussion

A large hurdle for HIV prevention in youth is inconsistent testing leading to an unknown number of undiagnosed cases. A lack of awareness of infection status can lead to rapid spread of HIV infection. Youth engage in health-risking behaviors that lead to an increased susceptibility to HIV transmission, yet only approximately 59% of HIV-positive youth are aware of their infection [3]. This study sought to survey a young population at increased risk for HIV acquisition and transmission to assess the potential of expanding the use of HIV self-testing in this group. While our study was limited in scope, several interesting conclusions can be made from our results.

We chose to survey youth whose demographics indicated that they were likely to be at an increased risk of STI and HIV acquisition because these youth are the group most likely to benefit from HIV screening. Choosing to do our survey at clinics offering sexual health services was one way to allow us to narrow our focus to this group. Analysis of the demographics of the survey participants confirmed that they included youth who had multiple sexual partners, a past history of sexual transmitted infections, and young men who had sex with men.

When this group of youth at higher risk for STI and HIV acquisition were questioned about their preferred HIV-testing location, the majority reported a preference for testing in the clinic setting compared to home HIV testing. We did find a significant difference in testing location preference between genders, with males more likely than females to say they preferred home testing, but overall, higher numbers of males still indicated that they preferred clinic-based testing (*p* = 0.031). The fact that eight participants did not specify a gender and two misinterpreted this question suggest that this statistical finding may not be very robust and should be studied further. No statistical difference was noted regarding testing location preference between youth with previous HIV testing, previous STI testing, or a history of previous STI. In addition, there was no significant difference by age in preferred location of HIV testing. 

Despite the fact that youth initially reported a preference for testing in a clinic, their responses to the Likert-scale questions indicate an openness to considering self-testing. Overall, all of the youth studied were receptive to the idea of HIV self-testing, with broad agreement with questions involving a willingness to do self-testing and strong disagreement with the idea of never doing self-testing. There was no statistically significant difference (*p* > 0.05) using the Likert-scale questions regarding attitudes about HIV self-testing at home between gender, previous HIV testing, previous STI testing, previous STI diagnosis, or history of HIV positivity. This suggests that broad categories of youth, particularly those at higher risk of HIV acquisition, could be targets for HIV self-testing initiatives. While the youth we surveyed were very familiar with STI and HIV testing in the clinical setting, with over 70% having had previous testing, only four youth had performed a home test, which likely contributed to their preference for clinic testing. With appropriate educational efforts, our results support the idea that home HIV testing could be another useful tool to increase HIV testing rates in youth and encourage repeat testing in those with behaviors that continue to put them at risk for HIV acquisition. 

There were several limitations to this study, including its exploratory nature. The data was gathered from a single geographical area, Tampa Bay, with potential for selection bias and not being representative of a larger population. The study population was not representative of the general population of US adolescents in terms of HIV-testing rate (72.9% tested vs. 9–15%), although it does represent a group that will benefit greatly from HIV screening; the results may not be generalizable to all youth. The responses were based on self-reporting data which are subject to recall and desirability biases. Other limitations are that the survey was not verified, some questions were not clearly understood by participants, and Likert questions could have been subject to both ceiling and floor effects. 

We propose a larger multi-institutional study to both improve the power of the study and validate our findings. Further research should examine the discrepancy between genders in preference for location of testing to determine if this is due to our small sample size and omitted responses or reflects a true gender difference. We also did not address the impact of cost of self-testing kits on attitudes towards HIV self-testing, which should be explored in future research. Further studies to add to our understanding of youth attitudes regarding self-testing and testing location preferences will help to provide a foundation to develop approaches to increase testing rates in this population. 

## Figures and Tables

**Table 1:** Characteristics of Patients Who Completed Survey.

Clinic Site	# Patients (%)
**Ybor Youth Clinic**	121 (90.98%)
USF Student Health	8 (6.02%)
St. Petersburg HIV Clinic	2 (1.50%)
CMS Lakeland HIV Clinic	2 (1.50%)
**Gender**	
Female	63 (47.37%)
Male	60 (45.11%)
No Answer	8 (6.02%)
Answered Sexual Orientation	2 (1.50%)
**Sexual Orientation**	
Heterosexual	78 (58.64%)
Bisexual	10 (7.52%)
Gay Female	9 (6.77%)
Gay Male	31 (23.31%)
Other	5 (3.76%)
**# Sexual Partners in Past Year**	
0−2	60 (45.11%)
3−5	44 (33.08%)
6−10	15 (11.28%)
> 10	6 (4.51%)
No Answer	8 (6.02%)
**History of Previous STI**	
Yes	53 (39.85%)
No	48 (36.09%)
Never Tested	31 (23.31%)
No Answer	1 (0.75%)

**Table 2:** Demographics and Preference for Testing Location.

	Home (*n* = 39)	Clinic (*n* = 90)	t or χ2	df	*p*	Effect Size
**Age**	20.49 (2.29) ^1^	21.27 (2.26) ^1^	t =−1.790	127	0.076	0.343
**Gender**			χ2 = 4.645	1	**0.031**	0.198
Female	14 (36.84%)	47 (58.02%)				
Male	24 (63.16%)	34 (41.98%)				
**HIV Test Previously**			χ2 = 0.217	1	0.641	0.060
No	9 (23.08%)	26 (28.89%)				
Yes	30 (76.92%)	64 (71.11%)				
**STI Test Previously**			χ2 = 0.079	1	0.779	0.025
No	11 (28.21%)	27 (30.68%)				
Yes	28 (71.79%)	61 (69.32%)				
**STI-Positive Previously**			χ2 = 0.002	1	0.968	0.004
No	14 (48.28%)	33 (47.83%)				
Yes	15 (51.72%)	36 (52.17%)				
**Ever Used Self-Test Kit**			-	1	1	0.019
No	37 (97.37%)	86 (96.63%)				
Yes	1 (2.63%)	3 (3.37%)				

^1^ Mean (standard deviation). Analysis excluded four youth without a preference for testing site and non-responders to the question.

## Data Availability

The data presented in this study are available on request from the corresponding author.
